# Crystallography of γ′-Fe_4_N formation in single-crystalline α-Fe whiskers

**DOI:** 10.1107/S1600576720005981

**Published:** 2020-06-12

**Authors:** Helge Schumann, Gunther Richter, Andreas Leineweber

**Affiliations:** a Institute of Materials Science, Technische Universität Bergakademie Freiberg, Gustav-Zeuner-Strasse 5, Freiberg, 09599, Germany; b Central Scientific Facility (CSF) Materials, Max Planck Institute for Intelligent Systems, Heisenbergstrasse 3, Stuttgart, 70569, Germany

**Keywords:** nitriding, whiskers, electron backscatter diffraction (EBSD), γ′-Fe_4_N, phenomenological theory of martensite crystallography (PTMC)

## Abstract

Gaseous nitriding of steel and iron can significantly improve their properties, for example corrosion resistance, fatigue endurance and tribological properties. In order to obtain a better understanding of the early stages of formation of the initial cubic primitive γ′-Fe_4_N, the mechanism and crystallography of the α–γ′ phase transformation was investigated under simplified conditions. Single-crystal α-Fe whiskers were nitrided at 823 K and a nitriding potential of 0.7 atm^−1/2^ for 20 min. The resulting microstructure and phases, as well as the crystallographic orientation of crystallites belonging to a particular phase, were characterized by scanning electron microscopy coupled with electron backscatter diffraction. The habit planes were investigated by single- and two-surface trace analysis. The α-Fe whiskers partly transform into γ′-Fe_4_N, where γ′ grows mainly in a plate-like morphology. An orientation relationship close to the rational Pitsch orientation relationship and {0.078 0.432 0.898}_α_ and {0.391 0.367 0.844}_γ′_ as habit planes were predicted by the phenomenological theory of martensite crystallography (PTMC), adopting a {101}_α_〈101〉_α_ shear system for lattice invariant strain, which corresponds to a {1 1 1}_γ′_〈1 12〉_γ′_ shear system in γ′. The encountered orientation relationship and the habit planes exhibit excellent agreement with predictions from the PTMC, although the transformation definitely requires diffusion. The γ′ plates mainly exhibit one single internally untwinned variant. The formation of additional variants due to strain accommodation, as well as the formation of a complex microstructure, was suppressed to a considerable extent by the fewer mechanical constraints imposed on the transforming regions within the iron whiskers as compared to the situation at the surface of bulk samples.

## Abstract 2

The crystallography and mechanism of the early stages of the formation of cubic primitive γ′-Fe_4_N in single-crystal α-Fe whiskers during gaseous nitriding were investigated by electron microscopy and electron backscatter diffraction. The α–γ′ phase transformation could be observed under simplified conditions owing to the lack of geometric constraints for the transformation.

## 1. Introduction   

**Figure 1:**
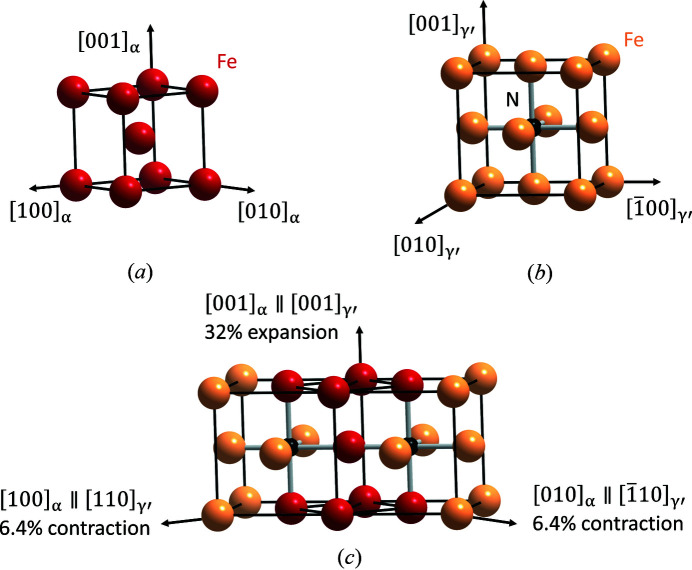
Crystal structures of (*a*) α-Fe (b.c.c. structure, *a*_α_ = 2.866 Å, space group 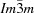; Straumanis & Kim, 1969 ▸) and (*b*) γ′-Fe_4_N (f.c.c.-like structure with N ordering making the translation lattice primitive, *a*_γ′_ = 3.790 Å, space group 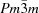; Jack, 1948 ▸). (*c*) Bain distortion sketched for the α →γ′ transformation.

The surfaces properties of a material can be significantly improved by a phase transformation, achieved, for example, by a thermochemical surface engineering process such as gaseous nitriding. Gaseous nitriding of iron and steel improves the tribological properties, the fatigue endurance and the corrosion resistance. Upon nitriding, an iron nitride compound layer is formed at the surface with a diffusion zone enriched with nitro­gen underneath (Somers, 2011 ▸; Mittemeijer & Somers, 2015 ▸). Gaseous nitriding is typically performed at temperatures between 773 and 853 K, and the formation of the compound layer on pure body-centered cubic (b.c.c.) α-Fe [Fig. 1 ▸(*a*)] has been shown to start with the nucleation and growth of cubic primitive γ′-Fe_4_N [Fig. 1 ▸(*b*)] as a stable phase. Additionally, during cooling or as a result of aging at temperatures lower than ∼523 K, body-centered tetragonal (b.c.t.) α′′-Fe_16_N_2_ can be formed as a metastable phase (Mittemeijer & Somers, 2015 ▸). In particular, the early stages of the formation of the γ′-Fe_4_N phase at the surface of the alloy are often crucial for the compound layer’s microstructure, because this typically is the first stage of compound-layer formation in low-alloy steel and iron.

In order to obtain more information about the crystallography of the nitride formation, the formation of b.c.t. α′′-Fe_16_N_2_ and the face-centered cubic (f.c.c.)-like γ′-Fe_4_N phase within the volume of α-Fe has been extensively studied for bulk-alloy material. To this end, N-enriched α-Fe was aged at temperatures lower than the typical nitriding temperatures, leading to precipitation of the γ′ phase due to the decreasing solubility of N in α-Fe with decreasing temperature (Koritke & Pitsch, 1964 ▸; Pitsch, 1961*b* ▸; Ferguson *et al.*, 1983 ▸; Booker, 1961 ▸; Dahmen *et al.*, 1987 ▸) (and of α′′-Fe_16_N_2_ if aging is performed at even lower temperatures^1^). The precipitates (assuming a quite small volume faction) are reported to be plates on ∼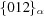 habit planes, which later join forming V-shaped particles, where the orientation and the growth direction depend on the orientation of the α-Fe parent phase. Similar microstructures have recently been reported in meteorites having an α-Fe–Ni (kamacite as a mineral) matrix containing γ′ plates (roaldite as a mineral) (Nolze & Heide, 2019 ▸). The combination of different γ′ variants^2^ has been attributed to accommodation of the strains imposed on the α-Fe matrix, as the transformation occurs by some shear process (see below) (Dahmen *et al.*, 1987 ▸). Another feature of the γ′ plates is striations parallel to the 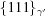 planes, which were attributed to a shear in the 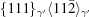 system accompanying the precipitation (Pitsch, 1961*b* ▸).

In contrast to reports of the γ′ precipitates developed upon aging of N-supersaturated α-Fe in the bulk, investigation of the direct formation of γ′ at the surface (Naumann, 1968 ▸; Inokuti *et al.*, 1975 ▸; Pulkkinen, 1982 ▸; Friehling *et al.*, 2001 ▸) of plate-like α substrates, grown under nitriding conditions, reveals deviations from the previously mentioned features. In particular, observations of nitride formation at the surface of polycrystalline samples reveal complex morphologies (*e.g.* leaf-like, needle-like or star-like) of γ′ in the early stages of nitriding. The nitride normally grows in a wedge-like manner into the α-Fe substrate.

Various orientation relationships (ORs) have been reported between the α and γ′ phases (irrespective of the location of formation of the latter phase), like near Kurdjumov−Sachs (KS) (Pitsch, 1961*b* ▸; Koritke & Pitsch, 1964 ▸), near Pitsch (Pitsch, 1961*b* ▸; Koritke & Pitsch, 1964 ▸; Dahmen *et al.*, 1987 ▸; Liu *et al.*, 2001 ▸, 2002 ▸) and near Nishiyama−Wassermann (NW) (Mehl *et al.*, 1934 ▸; Booker, 1961 ▸; Inokuti *et al.*, 1975 ▸), mainly on the basis of investigations with transmission electron microscopy of aged samples. The ideal rational versions of these ORs are summarized in Table 1 ▸. Additionally, Mehl *et al.* (1934 ▸) and Booker (1961 ▸) proposed an OR (Table 1 ▸) with 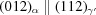 and 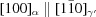.

Some important microstructural features of the α–γ′ transformation are similar to those of a martensitic transformation, *i.e.* a prominent habit plane, the presence of a characteristic slip in precipitates and the formation of surface relief (Pitsch, 1961*a* ▸). In that work it was shown that, for γ′ having formed as plate-like precipitates within α-Fe, the experimentally encountered habit plane and OR can be reconciled with the phenomenological theory of martensite crystallography (PTMC) (Pitsch, 1961*a* ▸; Otte & Massalski, 1958 ▸). Therefore, it was assumed that the formation of γ′ is mainly based on the motion of the iron atoms, and the nitro­gen atoms compulsorily move with them. Hence, the phase transformation requires N diffusion while the motion of the Fe atoms might correspond to a collective deformation like invariant plane strain in martensites (see below). That situation has been compared with that upon formation of bainite (Pitsch, 1961*a* ▸). It is, however, noted that in later work it was preferred to employ an invariant line approach (Dahmen *et al.*, 1987 ▸), which nevertheless gives a very similar description to using the PTMC approach in earlier work.

Our own research interest focuses on the mechanism and crystallography of phase formation beyond the formation of isolated precipitates, using the formation of γ′ at and below typical nitriding temperatures of 823 K as a model process. It turns out that the early stages of γ′ formation on bulk Fe surfaces lead to pronounced twinning of the γ′ grains (as also implied by earlier research; Liu *et al.*, 2002 ▸), indicative of accommodation of the transformation shear by formation of different variants (to be published), leading to complex microstructure and orientation gradients. In order to study γ′-Fe_4_N formation under simplified conditions, experiments on α-Fe (single-crystal) whiskers were performed.

## 2. Methods   

### 2.1. Sample preparation   

Single-crystal α-Fe whiskers were used as substrates for the nitriding experiments. The iron whiskers were produced by reduction of iron(II) chloride tetrahydrate (FeCl_2_·4H_2_O) powder in a fused-silica tube furnace under flowing hydrogen (Brenner, 1956 ▸). A corundum boat filled with 1–3 g of the powder was covered with an iron plate as growth substrate for the whiskers. The growth substrate was ultrasonically cleaned with ethanol and oxidized at 873 K in air prior to the growth process. The boat was loaded into a gas-flow quartz-glass tube (diameter: 40 mm) furnace at room temperature. The tube was evacuated (medium vacuum) and then purged in a first step with high-purity Ar (∼9 l h^−1^) during heating to 823 K with a heating rate of 15 K min^−1^. In a second step, the whiskers were grown by reduction of the iron(II) chloride tetrahydrate in flowing H_2_ (∼11 l h^−1^)/Ar (∼9 l h^−1^) at 823 K for 10 h. In order to obtain oxide-free whiskers, the reductive H_2_/Ar flow was kept constant during the cooling period (Yi *et al.*, 2004 ▸).

The growth substrates with the whiskers were nitrided in a laboratory chamber furnace in an NH_3_ and H_2_ gas mixture (300 l h^−1^) at 823 K and a nitriding potential of 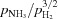 = 0.7 atm^−1/2^ for 20 min (*p*: partial pressures in the furnace gas mixture as measured in the furnace chamber and at the exhaust). Under these conditions, γ′-Fe_4_N is the only nitride phase that should be formed in metastable equilibrium with the gas (Lehrer, 1930 ▸; Göhring *et al.*, 2016 ▸). After nitriding, the samples were cooled by moving them into the cold zone of the furnace under process gas.

The cross sections of some unnitrided whiskers were investigated by optical-light microscopy (Neophot 30 microscope) and field emission (FE) scanning electron microscopy (SEM) and the surface before and after nitriding by SEM and electron backscatter diffraction (EBSD). EBSD measurements were conducted employing a Carl Zeiss LEO 1530 FE scanning electron microscope equipped with an Oxford Instruments HKL Channel 5 EBSD system and a JEOL JSM 7800F FE scanning electron microscope equipped with the EDAX Hikari Super EBSD system at an acceleration voltage of 20 or 30 kV. The employed step width during EBSD scanning was 50–100 nm.

For cross sections, the whiskers were removed with tweezers from the growth substrate, embedded in Struers PolyFast, ground and polished with Buehler MasterMet 2 colloidal silica suspension. EBSD was not performed on the cross sections of the whiskers owing to scatter of the orientation data, which could be attributed to, for example, damage during the sample preparation.

For EBSD measurements on the surface of the whiskers, they were removed with tweezers from the growth substrate and pasted onto a carbon-based, electrically conductive, adhesive disc on a specimen stub. Additionally, one whisker was pasted onto a small copper cuboid for a two-surface analysis. For that purpose, the tip of the whisker was fixed with conductive silver and the long side was aligned with one edge of the cuboid. The rectangular cross section of the cuboid allowed an exact tilt of about 90°.

To ensure that the whiskers are placed perfectly flat on the specimen stub, the fixed whiskers were viewed under different tilt and rotation angles in the scanning electron microscope. For an easy determination of the growth axis, the long axis of the whiskers was aligned along the **x** or **y** direction of the specimen stage (**x** 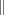 tilt axis of the specimen stage, **y** 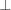 tilt axis of the specimen stage). For ease of use, only the longest whiskers were used for EBSD investigations. For trace analysis, only whiskers with the surface nearly parallel to the surface of the specimen stub were used. Note that the described procedure was applied to an individual whisker either only in the unnitrided state or only in the nitrided state.

### 2.2. EBSD   

EBSD data were analyzed using *HKL Channel 5* (Oxford Instruments, Abingdon, UK), the TSL *OIM* software (TexSEM Laboratories/EDAX, Mahwah, NJ, USA), *PTCLab* (Gu *et al.*, 2016 ▸) and the MATLAB toolbox *MTEX* (Bachmann *et al.*, 2010 ▸; Krakow *et al.*, 2017 ▸). In order to avoid the use of inaccurate measurements, data points with a mean angular deviation greater than 1°, or a confidence index lower than 0.2, were removed from the EBSD data. The data set was cleaned by removing small grains (<10 pixels), the unindexed or removed points being assigned to the closest grain and the phase of the closest measurement point. The grains were defined by using a threshold misorientation of 2°. Such a relatively small misorientation angle was chosen because the smallest misorientation angle between two variants for the encountered OR is about 4.2° (see Table 2 ▸). A constrained Laplacian smoothing was applied to the grain boundaries, in order to reduce the staircase effect (Hielscher *et al.*, 2019 ▸).

The growth axis and the facets of the iron whiskers were determined by analysis of the residual iron of the nitrided whiskers. Therefore, the orientation data obtained by EBSD were compared with the long axis and the edge of the whiskers in the SEM micrographs. The accuracy of the axis determination depends on the correct alignment of the whisker:^3^ typically an accuracy of absolute orientations of about 1° could be achieved (Susan *et al.*, 2012 ▸). The axis and the facets were assigned to the best-matching low-index axis directions and face planes, in accordance with axes and faces reported in the literature (Coleman, 1958 ▸; Yi *et al.*, 2004 ▸; Chikaura & Nagakura, 1972 ▸; Bojarski & Surowiec, 1979 ▸; Gardner, 1978 ▸).

The experimentally determined misorientation at the α–γ′ boundary and the ORs were described by rotational axis–angle pairs. Therefore, a number of crystallographically related, mathematically equivalent solutions exist. Owing to the crystal symmetry, an axis–angle pair exists that exhibits the smallest rotation angle about a rotation axis – called disorientation (Krakow *et al.*, 2017 ▸; Randle & Engler, 2010 ▸). In view of the scatter of orientation data, agreement of experimental misorientation data with different orientation relationships was assessed by a misorientation angle distribution for the α–γ′ boundary misorientations, representing the deviation of the experimental data from the theoretical ORs in axis–angle space (Randle & Engler, 2010 ▸). Note that correlated misorientations along the α–γ′ boundaries were used for the determination of the orientation relationship.

For the identification of the γ′ variants, the mean orientation of α-Fe and, for each whisker, a fixed coordinate system were assigned (see Section 4). In order to prevent inaccurate α-Fe orientations due to slight orientation variations, only orientations around the analyzed area were used. The γ′ variants of the different ORs have been calculated according to the transformation rules for the α-Fe parent phases, using the numbering scheme adopted from the list of KS variants given by Morito *et al.* (2003 ▸) (see Table S1 of the supporting information). Each of the current variants is assigned the same variant number as the orientationally closest KS variant.

For analysis of the experimental orientations of the γ′ phase in a (region of a) whisker, theoretical poles expected for γ′ of a certain OR with respect to α were plotted in a standard pole figure for the α-Fe parent crystal. Subsequently, the theoretical template pole figures were superimposed with the experimentally encountered pole densities of γ′ with respect to the mean orientation of the α-Fe parent whisker.

A direct determination of a habit plane is possible by a two-surface trace analysis (Rowlands *et al.*, 1968 ▸; Fearon & Bevis, 1974 ▸; Zhang & Kelly, 2009 ▸; Tahara *et al.*, 2017 ▸). For that purpose, the directions corresponding to the traces of the habit plane are measured on two corresponding surfaces, where the orientation of the two surfaces is known. The habit plane normal is calculated from the cross product of the two vectors.

Once the variants were assigned by the superposition of the pole figures of the experimental orientations and the template pole figure for the calculated variants, it was possible to compare the predicted habit planes with the experimentally observed trace, *i.e.* the line where the habit plane intersects the whisker surface. For that purpose, the calculated habit planes predicted (see Section 2.3) for the orientation variant encountered were plotted in a stereographic projection, and the projection was rotated according to the average orientation of the α-Fe matrix. In this way, the positions of the habit planes belonging to a specific variant were obtained. The experimentally obtained α and γ′ poles of the (*hkl*) describing the habit planes should match with the habit planes predicted by PTMC, and the corresponding plane traces must coincide with the α–γ′ interface for the variant encountered. In this way a clear identification of the variants that are consistent with the habit planes was possible.

### 2.3. Predictions using the PTMC   

The basic equation of the PTMC is
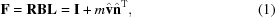
where 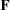 is the matrix describing the macroscopic deformation from the parent to the product phase, corresponding to an invariant plane strain (IPS). In the middle part of equation (1), **F** is expressed as the matrix product of a rigid-body distortion 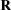, the (symmetric) Bain distortion 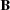 and the lattice invariant strain 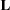 (LIS). The right-hand side of equation (1) corresponds to a general formulation of the IPS with a unit vector 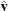 in the direction of the shape deformation, a unit vector 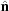 normal to the invariant habit plane and the magnitude of the strain *m*. As already indicated by the middle part of equation (1), the overall deformation associated with the transformation process is artificially divided into three successive steps expressed in terms of a multiplication of three matrices describing the particular steps. The sequence of these steps adopted here is motivated by computational simplicity and assumes that the LIS is followed by the Bain distortion and then by the rotation.

The LIS can be described as a shear on a plane 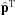 (row vector) along the shear direction 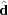 (column vector) with a magnitude of shear 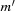, leading to
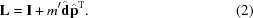
The simple shear requires 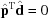, which is not the case for 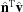 in equation (1). The Bain distortion 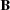 homogeneously strains (stretches) the arrangement of iron atoms corresponding to the b.c.c. α structure towards the f.c.c. structure of the iron atoms in the γ′ phase, and the Bain (lattice) correspondence matrix 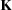 relates the body-centered unit cell of the tetragonally distorted α lattice to the quasi-face-centered-cubic unit cell of the γ′ lattice [Fig. 1 ▸(*c*)]. The product **BL** describes a deformation with an undistorted (habit) plane, which is, however, still rotated between the α and the γ′ lattice and which already implies an invariant line. With a given 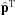 and 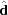 the magnitude of shear 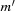 is obtained by solving the characteristic equation (the two vertical bars denote the determinant)
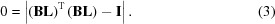
The invariant plane is achieved by a final rigid body rotation 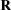 of γ′. In doing so one obtains one or several solutions for *m*′, the orientation relationship 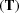, the habit planes 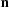 and the overall IPS 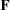 (Schumann, 1979 ▸, 1981 ▸; Nishiyama, 1978 ▸).

## 3. PTMC predictions   

In order to allow predictions with the PTMC, different input parameters are necessary, which involve the crystal structures, the lattice parameters, the atomic correspondence between the two phases and some assumptions about the character of the lattice invariant shear (Zhang & Kelly, 2009 ▸). The input parameters for the calculations of variant 1 are summarized in Table 3 ▸. For the lattice invariant shear a 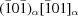 slip system has been adopted, defining 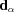 = **d** and 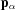 = **p**. The correspondence matrix **K** between the γ′ and the α lattices yields for the slip plane normals 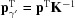 and for the lattice directions 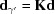 (Schumann, 1979 ▸; Nishiyama, 1978 ▸), corresponding to the type of 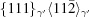 slip system already used by Pitsch (1961*a* ▸). With the lattice parameters, the Bain distortion 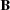 leads to an expansion of about 32.2% along 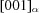 and a compression of about 6.4% along 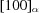 and 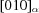 [*cf*. Fig. 1 ▸(*c*)]. Application of symmetry to **p**, **d** and **K** leads to the input parameters necessary for the calculation of the remaining variants.

The results of the calculations (Table 4 ▸) comply with the results obtained by using the *PTCLab* (Gu *et al.*, 2016 ▸) software for the corresponding input parameters. The misorientation between two ORs was calculated from the transformation matrices, by which means a minimal rotation angle was calculated for each specific variant pair (Nolze, 2008 ▸). In order to find for a given OR variant the smallest misorientation (disorientation) angle and the rotation axis, the crystal symmetry must be considered, which was done with the help of a corresponding tool in the software *MTEX*.

**Figure 2:**
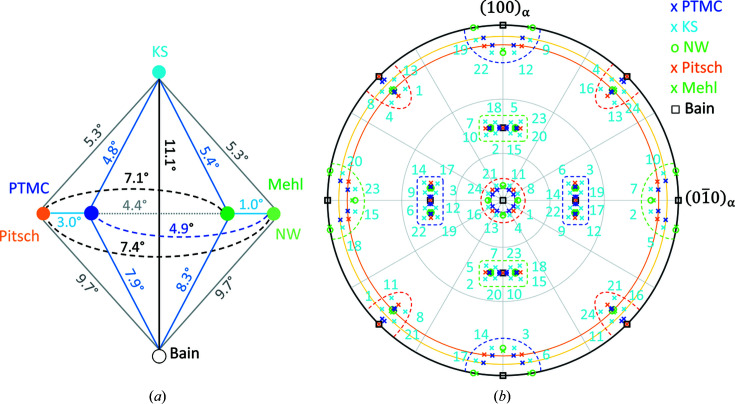
Comparison of the rational ORs with the calculated PTMC OR. (*a*) Rotation angles between the different ORs. (*b*) Stereographic projection of the orientation variants represented as {100}_γ′_ pole figures projected on 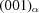. For better clarity, only the KS variants are numbered (*cf*. Table S1 in the supplementary materials). The Bain zones are marked with dashed curves (B1: red; B2: green; B3: blue) and the surface groups with solid circles (S1: magenta circle connecting blue crosses at the center; S2: dark orange; S3: yellow) (*cf*. Table 2 ▸). Note that the PTMC OR deviates less from the Bain OR than the other rational ORs.

The OR predicted by PTMC deviates only by 0.8° from that predicted by Pitsch (1961*a* ▸) by PTMC calculations. That difference is caused by the different lattice parameter values adopted for the calculations. Note that the presently predicted OR deviates by about 3° from the rational Pitsch OR [Fig. 2 ▸(*a*)]. The calculated habit planes are close to the calculated and/or experimentally observed ∼{0.08 0.44 0.89}_α_ (Pitsch, 1961*b* ▸,*a*; Koritke & Pitsch, 1964 ▸), {049}_α_ (Dahmen *et al.*, 1987 ▸) or {012}_α_ (Mehl *et al.*, 1934 ▸; Booker, 1961 ▸) and {112}_γ′_ (Mehl *et al.*, 1934 ▸; Booker, 1961 ▸; Pitsch, 1961*a* ▸,*b* ▸; Koritke & Pitsch, 1964 ▸) planes, which were reported as habit planes, with deviation angles of ∼0.6, 4.8, 4.6 and 3.0° relative to the habit planes predicted by PTMC in this work.

An overview of all variants is given stereographically in Fig. 2 ▸(*b*)] The variants can be grouped into three different Bain zones B1–B3 (depending on their lattice correspondence), six different shear groups SP/SD1–SP/SD6 (there are six different 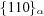 slip planes, each with one 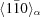 slip direction) and three different surface groups S1–S3 (depending on the modification of the surface after lattice invariant shear; see Sections 5.1.3 and 6). Thus, variant 1 belongs to B1, SP/SD1 and S1. For the α parent phase shown in Fig. 2 ▸(*b*), the variants belonging to B1 exhibit approximately a {100}_γ′_ plane parallel to the exposed (001)_α_ face, whereas the variants belonging to B2 and B3 exhibit approximately a {110}_γ′_ plane, represented in the inverse pole figure (IPF) maps of the present paper by red coloring for B1 group variants and green coloring for B2/B3. The approximate character of the exposed (*hkl*) is caused by the lattice invariant shear.

## 4. Whiskers prior to nitriding   

Whiskers grew directly on the growth substrate but also in the corundum boat supporting the substrate. Polishing or cleaning of the surface of the growth substrate prior to the reduction treatment suppressed the growth of the whiskers. The best results for whisker growth were obtained when the growth substrate surface was oxidized prior to conducting the reduction experiment.

**Figure 3:**
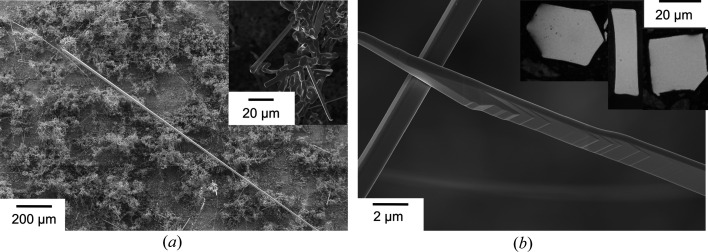
Surface of the growth substrate after whisker growth. (*a*) Whiskers of different sizes and intergrown whiskers (inset) and (*b*) growth steps on the surface of a whisker. The inset shows optical micrographs of cross sections of iron whiskers.

The whiskers show a vast range of sizes and lengths of up to several millimetres with diameters of up to 50 µm [Fig. 3 ▸(*a*)]. All whiskers (including the nitrided ones) investigated by SEM/EBSD on the carbon pads appeared to expose (001)_α_ facets, whereas the whisker (growth) axes are either [100]_α_ or [110]_α_. These specific plane and direction indices are used as a frame of reference for indexing the α phase in the following sections. The preferential growth of whiskers with [100]_α_ growth axis was previously attributed to the nucleation on iron oxide (Cabrera *et al.*, 1958 ▸). The cross sections are compatible with a square, rectangle or hexagonal profile and belong to the commonly observed growth forms (Yi *et al.*, 2004 ▸; Chikaura & Nagakura, 1972 ▸; Coleman, 1958 ▸; Bojarski & Surowiec, 1979 ▸; Gardner, 1978 ▸).

**Figure 4:**
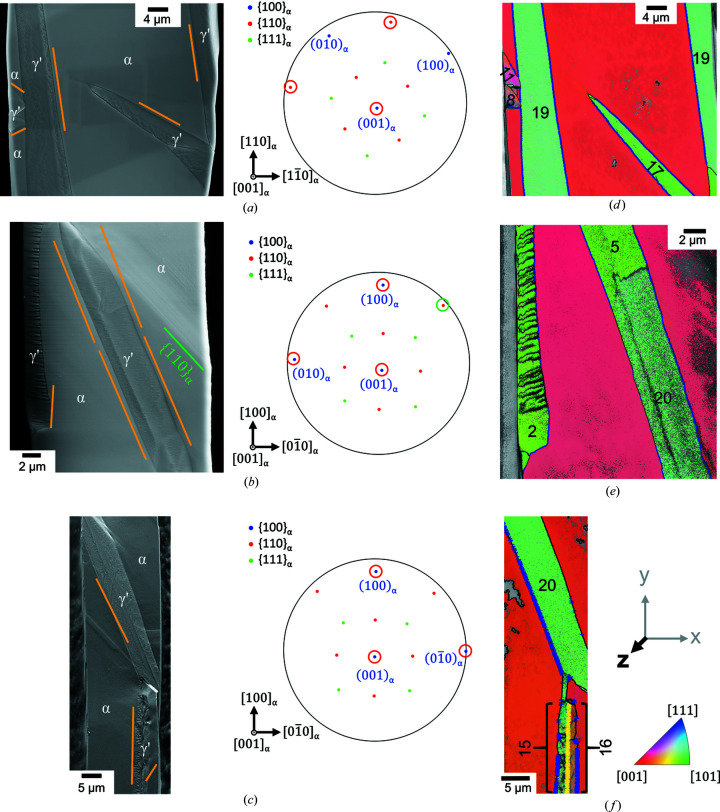
(*a*)–(*c*) SEM images of three different nitrided whiskers and the corresponding mean orientation of the residual iron indicated in a stereographic projection. All whiskers exhibit 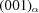 surfaces. The orange lines mark the plane traces of the habit planes predicted for the particular variant. The growth axis and the possible facets are marked by red circles. (*d*)–(*f*) Superposition of the band contrast maps with the IPF maps of the nitrided iron whiskers corresponding to (*a*)–(*c*). The α–γ′ interface is marked with a blue line, if the deviation from the PTMC OR is ≤2°. The numbers denote the identified PTMC variants (see Fig. 2 ▸). The yellow line in (*f*) marks the Σ3 twin boundary between two γ′ variants, if the deviation is also ≤2°.

Separate results on crystallographic characterization of unnitrided whiskers are not shown here, because corresponding features are equally well discerned from the untransformed regions of the nitrided whiskers (see Fig. 4 ▸).

## 5. Analysis of nitrided whiskers   

As visible in the SEM images [Figs. 4 ▸(*a*)–4 ▸(*c*)], the γ′ and α phase are clearly distinguishable by backscattering contrast and an unambiguous distinction was possible by EBSD. The whiskers were, in all cases, only partially transformed and the remaining α-Fe showed mainly a smooth surface. Rarely observed striations in the remaining α-Fe across the complete whisker may arise from slip on {110}_α_ [Fig. 4 ▸(*b*)] due to stresses during the preparation, *i.e.* unrelated to the formation of γ′.

**Figure 5:**
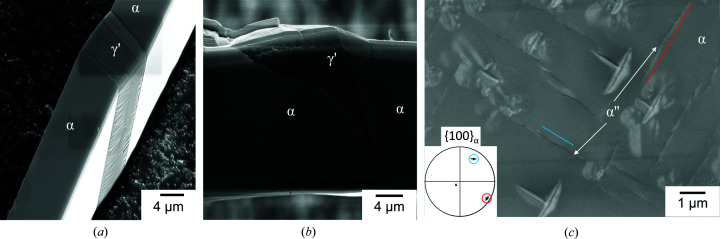
SEM images of the surface of nitrided whiskers. (*a*) Plate-like iron nitride with kinking and (*b*) wedge-like iron nitride with surface upheaval. (*c*) The striations belong to α′′-Fe_16_N_2_ formed on {100}_α_ planes as indicated with the plane traces. The inset shows the {100}_α_ pole figure with markings of the plane traces.

The γ′ phase was predominantly grown with a plate- or tapered-plate-like morphology. In that way, it often grew through the whole width of the whisker [Fig. 5 ▸(*a*)]. Additionally, γ′ occasionally grew along the edge of the whisker, usually with a rough surface [Fig. 4 ▸(*b*)].

Mostly, the formed γ′ could be divided into three different main categories:

Category (i): γ′ growing across the whisker with a straight α–γ′ interface, predominantly free of low-angle grain boundaries

Category (ii): γ′ growing along the edge of the whisker, often with many low-angle grain boundaries within

Category (iii): γ′ variant pairs with (coherent) Σ3 {111}_γ_ twin boundaries

It was anticipated that category (i) γ′ complies with the PTMC predictions in the cleanest fashion. Characteristic features of the γ′ phase were striations on the surface. Kinking [Fig. 5 ▸(*a*)] and surface upheavals [Fig. 5 ▸(*b*)] were observed, which arise from the volume expansion due to the nitro­gen absorption and/or the shear during the phase transformation. Another feature observed on the nitrided iron whiskers was plate-like features along {100}_α_ plane traces [Fig. 5 ▸(*c*)], which were attributed to α′′-Fe_16_N_2_ formed during the cooling process. α′′-Fe_16_N_2_ is known to form as plates on the {100}_α_ planes with a 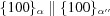, 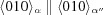 OR (Jack, 1951 ▸; Liu *et al.*, 2001 ▸; Koritke & Pitsch, 1964).

### 5.1. Category (i) plates   

#### 5.1.1. Orientation relationship   

**Figure 6:**
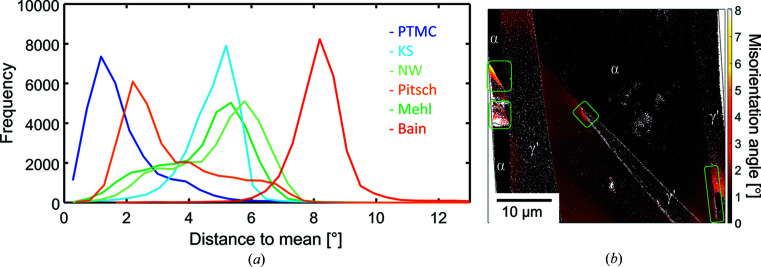
(*a*) Frequency distribution of occurrence of α–γ′ ORs of 119 grains. (*b*) Intergranular misorientation with respect to the mean orientation. The green rectangles mark areas with larger misorientations.

The frequency distributions of the classical rational ORs and the PTMC OR of >30 000 data point pairs pertaining to 119 γ′ grains from 19 maps are shown in Fig. 6 ▸(*a*). The PTMC solution fits best with the experimentally measured OR with a maximum in the frequency distribution function for disorientation at ∼1.4°. Among the classical ORs listed in Table 1 ▸, the Pitsch OR shows the best match with a peak at ∼2.4°. The KS, NW, Mehl and Bain ORs show a clearly larger deviation from the experimental data of about 5–8°. Hence, the predicted PTMC OR shows a better agreement than the Pitsch OR and the other prominent rational ORs. In fact, the rotation of the PTMC OR away from the Bain OR is predicted and found to be smaller than that for the other, more prominent ORs. This good agreement confirms the predictive power of the PTMC calculations.

In Figs. 4 ▸(*d*)–4 ▸(*f*) the α−γ′ phase boundaries have been highlighted in blue if there is a deviation of ≤2° of the experimental OR from the PTMC OR. The boundaries not matching the PTMC OR within 2° contribute to the shoulders of the peak of the frequency distribution and occur mainly in the areas where α-Fe deviates from the assigned average orientations [Fig. 6 ▸(*b*)]. The source of the orientation deviation is probably the interaction of strain fields around different γ′ variants.

#### 5.1.2. Habit plane   

The γ′ phase shows different orientations and variants, but common to most plates observed is an approximate {110}_γ′_ face parallel to the exposed (001)_α_ face, as shown in the IPF maps in Figs. 4 ▸(*d*)–4 ▸(*f*). Consequently, these variants are assignable to the Bain zones B2 or B3. Therefore, the more frequent occurrence of {110}_γ′_ faces can probably be attributed to the larger number of possible variants (16 variants for B2 and B3 instead of eight for B1). The very good fulfillment of the PTMC OR by the variants of category (i) and the well developed, straight α–γ′ interfaces enabled a relatively straightforward comparison of the predicted habit planes with the experimentally observed ones.

**Figure 7:**
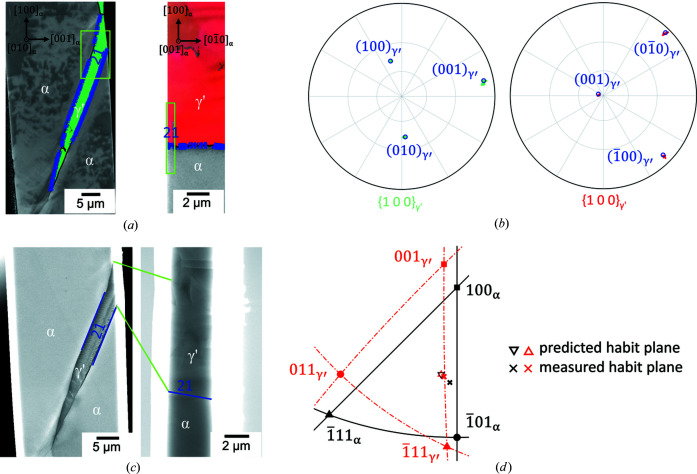
(*a*) IPF maps of the two surfaces of a nitrided whisker. The green rectangles mark the areas used for the (*b*) {100}_γ′_ pole figures. The blue circles in the {100}_γ′_ pole figures indicate the {100}_γ′_ poles of the appropriate PTMC variants. The α–γ′ interface in the IPF maps is marked with a blue line, if the deviation from the PTMC OR is ≤2°. (*c*) SEM micrographs of the two surfaces. The green lines mark the related surfaces in the SEM micrographs. (*d*) Habit plane locus for the PTMC prediction and the experimentally measured habit plane (black: α-Fe; red: γ′-Fe_4_N). The γ′ orientation corresponds to the identified PTMC variant with respect to α. The habit plane trace corresponding to the PTMC variant is marked with a blue line in the SEM micrographs.

Fig. 7 ▸(*a*) shows exemplarily a nitrided whisker investigated by a two-surface analysis, where the whisker is tilted about 90° around the long side of the whisker (*y* axis). The {100}_γ′_ poles of the best-matching variant (variant 21) are marked by blue circles in Fig. 7 ▸(*b*). The {100}_γ′_ poles of the assigned variant show for both surfaces a very good agreement with the experimentally observed {100}_γ′_ poles. As expected, the poles of the γ′ phase are rotated about 90° around the *y* axis and the same γ′ variant was unambiguously assigned on the basis of the EBSD data collected from both surfaces.

For the two-surface trace analysis [Fig. 7 ▸(*c*)], the habit planes given by the cross product of the determined vectors along the α–γ′ interface are 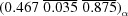 and 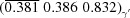, taking into consideration the mean orientation of α and γ′. These planes show a relatively small deviation of about 3.4 and 1.0° to the 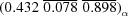 and 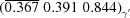 habit planes predicted for the identified PTMC variant. The small deviations can be attributed to measurement errors like the measurement uncertainty of the trace determination, small deviations of the whisker alignment or discrepancies of the orientation determination. The triangle stereographic projection of the habit plane locus reveals that the experimentally determined α and γ′ planes are in accordance with the calculated habit plane [Fig. 7 ▸(*d*)]. Furthermore, the trace of the habit plane predicted from the PTMC results for the orientation variant encountered shows a very good agreement with the observed trace of the α–γ′ interface.

**Figure 8:**
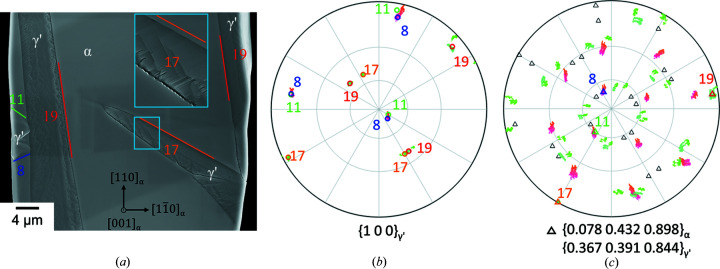
(*a*) SEM micrograph of the surface of the nitrided iron whisker with the habit plane traces corresponding to the PTMC variants. (*b*) {100}_γ′_ pole figure of the γ′-Fe_4_N phase and corresponding variant numbers from the IPF map shown in Fig. 4 ▸(*d*). (*c*) Superposition of the pole figures of the habit planes (mean orientation of the α region, experimental data of the γ′ region).

These results were verified by the single-surface trace analysis of different variants, exemplarily shown in Fig. 8 ▸. The poles of the calculated habit planes as well as the corresponding plane traces show an excellent match with the experimental data. So, the habit plane was clearly identified.

However, in the case of the tapered plate of variant 17 [Fig. 8 ▸(*a*)] the predicted habit plane shows a deviation for the lower part of the plate. The inset shows the α-γ′ interface in more detail, revealing a step-like interface, whereas the upper, well matching part of the plate exhibits a straight interface and a very good agreement with the predicted habit plane.

#### 5.1.3. γ′ variants related by low-angle grain boundaries   

**Figure 9:**
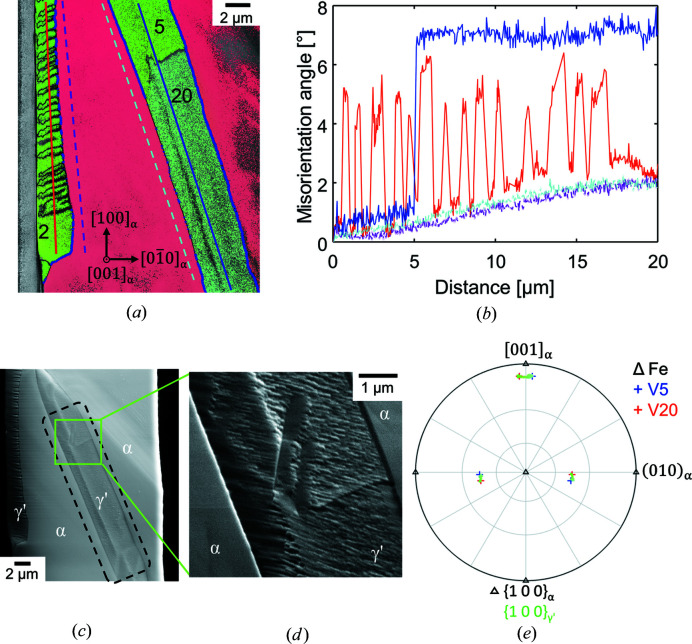
(*a*) IPF map of a nitrided iron whisker. The α–γ′ interface is marked with a blue line, if the deviation from the PTMC OR is ≤2°. The lines mark the positions and the directions of the (*b*) point-to-origin misorientation profiles in the γ′ (red and blue) or α (magenta and cyan) phase (measurement direction: downwards). (*c*) SEM micrograph of (*a*). The green rectangle marks the enlarged area [(*d*), SEM forward scatter detector micrograph] of the low-angle grain boundary between two PTMC variants. (*e*) Superposition of the {100}_γ′_ pole figure of the area marked by the black dashed line in (*c*) with the stereographic projection of the {100}_γ′_ poles of the corresponding variants. For a better overview the α-Fe orientation, and accordingly the orientation of the γ′ variants, was rotated into standard orientation with respect to α.

Usually, γ′ belonging to category (i) comprises one single PTMC variant with a straight α–γ′ interface plane parallel to the corresponding habit plane [Figs. 4 ▸(*a*) and 4 ▸(*c*)]. In the special case of the example shown in Fig. 4 ▸(*b*) and Fig. 9 ▸ two PTMC variants (5 and 20) with a straight α–γ′ interface are separated by a low-angle grain boundary. Both variants belong to B2 and S2, but they differ in the SP/SD group. The measured misorientation angle between the variants is approximately 6.5° [Fig. 9 ▸(*b*)], which is in relatively good agreement with the theoretical misorientation angle of about 7.7° of the corresponding PTMC variants (see Table 2 ▸). The small deviation can be attributed to, for example, the small orientation change of the α-Fe phase of approximately 2°, measurement inaccuracy or/and the orientation change within the γ′ grains towards the grain boundary. The two variants are mirror related across the (010)_α_ plane, or alternatively by a rotation of 180° around the [001]_α_ axis [Fig. 9 ▸(*e*)].

The 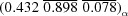 habit plane of variant 20 and the 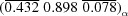 habit plane of variant 5 differ only slightly from each other (∼9°), and the plane trace is indistinguishable because the angle between the traces of the predicted habit planes is 0° on the (001)_α_ surface. Fig. 9 ▸(*d*) shows the surface of the transition zone between the two variants in more detail and a change of the surface features is visible.

#### 5.1.4. Surface striations   

**Figure 10:**
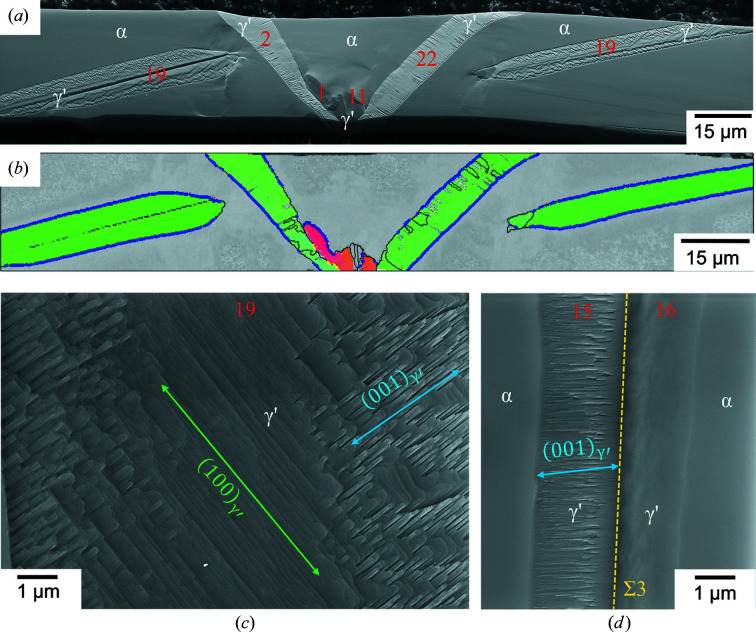
(*a*) SEM micrograph of a nitrided whisker including several variants that exhibit faceting. The numbers denote the appropriate PTMC variants. (*b*) Superposition of the band contrast map with the IPF map of the nitrided iron whiskers corresponding to (*a*). The α–γ′ interface is marked with a blue line, if the deviation from the PTMC OR is ≤2°. The yellow line marks the Σ3 twin boundary between two γ′ variants, if the deviation is also ≤2°. Magnified area of the SEM micrographs of (*c*) Fig. 4 ▸(*a*) and (*d*) Fig. 4 ▸(*c*), showing a detailed view of the surface features for different variants. The numbers denote the appropriate PTMC variants and the green and blue arrows mark traces of {100}_γ′_ planes.

The γ′ regions frequently exhibit characteristic striations on the surface. Depending on the surface group of the variants (Table 5 ▸), the γ′ region exposes different crystallographic planes to the surface as determined by the Bain group and by the type of LIS relative to the surface.^5^ Referring to this, Figs. 10 ▸(*a*) and 10 ▸(*b*) show the surface of a nitrided whisker with different variants belonging to the three different surface groups (color in the IPF maps: S1 – red, S2 – green, S3 – green). It appears that these striations occur for surface group S2 and S3 exposing exactly or approximately {110}_γ′_ planes parallel to the surface, whereas they are virtually absent for S1 exposing approximate {100}_γ′_ planes.

A detailed view of the surface features of variant 19 belonging to the whisker of Fig. 4 ▸(*a*) is shown in Fig. 10 ▸(*c*). The striations are parallel to (001)_γ′_ plane traces and these traces are parallel to the 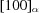 axis of expansion for the corresponding γ′ variant [*cf*. Fig. 1 ▸(*c*)]. Besides the aforementioned striations, additional striations occur occasionally, these less pronounced striations being oriented in another direction. A good correspondence between these striations and the (100)_γ′_ plane traces was determined, with the striations pointing approximately towards the 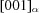 axis of contraction for the respective variant. For the sake of completeness, it should be mentioned that a good agreement with other plane traces, *e.g.* {110}_γ′_, was also determined. Our preference for the {100}_γ′_ plane traces is based on the explanation given in Section 6.

A further characteristic feature is a straight line at the center of variant 19, showing some similarity to a midrib plane, which is attributed to the first part of plate formation in the case of, for example, martensite formation in Fe–Ni alloys (Patterson & Wayman, 1966 ▸).

### 5.2. Categories (ii) and (iii)   

Especially in the case of the variants of category (ii), the γ′ grains often break up into subgrains separated by low-angle grain boundaries with a rotational angle of about 4.8°, and consequently more frequently exhibit a deviation from the PTMC OR. It is conspicuous that in the case of the category (ii) variants the low-angle grain boundaries can appear periodically [Figs. 9 ▸(*a*) and 9 ▸(*b*)]. No clear explanation of this feature has been given.

Variants with {100}_γ′_ approximately parallel to the exposed (001)_α_ face always belong to B1. In our investigations, the B1 types often pertain to category (iii) and occur in a twin relation with other variants with a coherent Σ3 {111}_γ′_ twin boundary between the two variants (incompatible with the PTMC OR α–γ′). The variants of category (iii) also show larger deviations from the ideal PTMC OR.

In accordance with the results of the deviation from the PTMC OR, the largest deviation between the calculated habit plane traces and the observed interface was encountered for the variants belonging to category (ii) and category (iii) [*cf*. Figs. 4 ▸(*b*) and 4 ▸(*c*)]. The formation of different variants and orientation gradients leads to deviations from the predictions obtained by PTMC.

With regards to the surface striations, a detailed view of the surface features of variants belonging to category (iii) for the whisker of Fig. 4 ▸(*c*) is shown in Fig. 10 ▸(*d*). The striations are also clearly visible on variant 15 with a (101)_γ′_ surface, where the pronounced striations are again parallel to the (001)_γ′_ plane traces and point approximately towards the 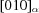 axis of expansion. In contrast, no pronounced striations are visible on the (100)_γ′_ surface of variant 16.

## 6. Discussion   

In the case of γ′ of category (i) the formation of self-accommodating variants, in order to reduce transformation strains, was avoided and the γ′ phase usually formed single variant plates. The experimental ORs and habit planes are in excellent agreement with the predictions obtained by the PTMC. Thus, the early stages of the α–γ′ transformation described for the direct growth of γ′ in α-Fe single-crystal whiskers during gaseous nitriding are comparable to the description of the α–γ′ transformation given for bulk-aged N-supersaturated α-Fe (see Pitsch, 1961*a* ▸; Dahmen *et al.*, 1987 ▸; Otte & Massalski, 1958 ▸). This is also shown by the mirror-related variants described in Section 5.1.3. This type of mirror-related variant was previously observed by Dahmen *et al.* (1987 ▸) in the case of nitride formation in bulk-aged samples, in which the two variants form one of the plates of the V-shaped precipitates without a pronounced strain accommodation. The formation of the second plate including the variants necessary for the strain accommodation was avoided owing to the reduced geometrical constraints of the single-crystal α-Fe whiskers.

As already described in Section 2.3, a deformation with an undistorted (habit) plane (invariant plane strain) can be obtained if Bain distortion and rotation are accompanied by a lattice invariant strain, where the same amount of LIS could be achieved by twinning or regular slip (Zhang & Kelly, 2009 ▸). In the case of bulk-aged N-supersaturated α-Fe, stacking faults within the precipitates grown at ∼573 K were formed because of slip (Dahmen *et al.*, 1987 ▸; Pitsch, 1961*b* ▸). The LIS considered there (and here) was 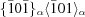, which corresponds to a 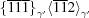 shear in γ′ being able to generate the stacking faults observed. The magnitude of shear 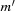 calculated by PTMC amounts to 0.132, which corresponds to a shear angle of about 7.5°. This means that for the 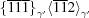 slip system slip takes place on every fifth to sixth slip plane (Pitsch, 1961*a* ▸). Dahmen *et al.* ( 1987 ▸) reported quite regularly spaced stacking faults on every sixth 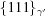 plane for precipitates formed at 623 K, and the periodically faulted structure formed was described as an 18H polytype. Our own investigations with X-ray diffraction performed on iron plates nitrided under the same conditions as the whiskers (to be published), and also results from the literature (Malinov *et al.*, 2001 ▸), yield narrow Bragg peaks indicative of almost perfect γ′, which contradicts the presence of stacking faults with densities as mentioned above. From this, we conclude that the γ′ regions generated in the whiskers upon nitriding at 823 K are also largely free of stacking faults. It is easily imaginable that stacking faults initially formed during the transformation have been annihilated by coalescence and/or annihilation of the partials, as described, for example, for Fe–30Mn–6Si shape memory alloy (Jiang *et al.*, 1997 ▸).

The observed surface striations of the γ′ surface can be attributed to surface faceting. Faceting occurs if the surface energy of a crystal surface can be reduced by ‘dissociation’ into surface planes of a lower energy, if the resulting increase in surface area is overcome by a reduction of the specific surface energy (Gjostein, 1963 ▸; Hermann, 2017 ▸). In accordance with the experimental observations, no faceting is expected for the {100}_α_ facets of the remaining α-Fe phase, since {100}_α_ planes are low-energy Wulff planes of α-Fe in contact with vacuum or H_2_ (Sundquist, 1964 ▸; Kovalenko *et al.*, 2016 ▸). The relative surface energies for different (*hkl*) surfaces are unknown for γ′, although energies for (only) {100}_γ′_ have been calculated by first-principles methods (Shi *et al.*, 2012 ▸). According to the broken bond model (Mackenzie *et al.*, 1962 ▸; Sang & Miller, 1971 ▸) with bond energies (actually energy increments) ∊ for an Fe−N bond (length 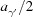) and η for an Fe−Fe bond (length 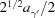), contributions due to these bonds yield to the specific surface energy for a given {*hkl*}_γ′_ as listed in Table 6 ▸ for the three lowest-index surfaces. Without the Fe−N bond contribution, the result agrees with the case for a pure f.c.c. structure (like γ-Fe), with {111}_γ′_ being the lowest-energy surface but where {100}_γ′_ with only a 15% higher specific surface energy also contributes to the Wulff shape (being a truncated octahedron; Sundquist, 1964 ▸). The contribution due to Fe−N shows an anisotropy, which strongly favors the {100}_γ′_ faces as compared to {110}_γ′_ and {111}_γ′_. A combination of these two contributions makes it probable that for γ′ the {100}_γ′_ face will dominate the Wulff shape. An exact or close-to {110}_γ′_ face having formed by transformation for surface groups S2 and S3 from an (001)_α_ face will then probably facet into two {100}_γ′_ faces. In the case of surface group S2 [adopting an integer version of the (*hkl*) from Table 5 ▸]
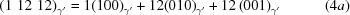
or for S3, where the surface (*hkl*) is exactly {110}_γ′_,
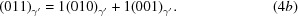



The stoichiometric coefficients then indicate the relative surface areas of these planes, suggesting that in both cases a pair of (010)_γ′_/(001)_γ′_ planes dominates a correspondingly faceted surface, intersecting at a 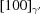 axis approximately or exactly in the plane. In the case of surface group S3 there is, indeed, only one type of dominant direction of the surface striations. In the case of S2, a minor 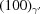 type of facet also occurs, which intersects the other two planes along 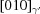 and 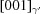. This direction projected onto 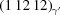 gives very similar directions. Hence, it appears plausible that one major and a single minor direction exist for the surface striations [see *e.g.* Fig. 10 ▸(*c*)].

In the case of surface group S1, the surface is already oriented close to the low-energy 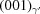, and hence the driving force for faceting will be very small. Accordingly, no visible striations indicating faceting are encountered.

## 7. Summary and conclusions   

The morphology and crystallography of γ′-Fe_4_N in α-Fe whiskers, formed directly by gaseous nitriding at 823 K, were examined by means of SEM coupled with EBSD. As starting material, two types of iron whiskers, with 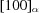 and 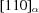 growth axis and 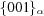 and 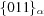 facets, were used.

During gaseous nitriding, α-Fe is partly transformed into γ′-Fe_4_N, in which the γ′ phase could be grouped into three main categories. The crystallographic nature of the γ′ formation could be described by the PTMC. The orientation relationship and the habit planes obtained by 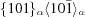 shear, corresponding to 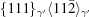 shear, show excellent agreement with the experimental data. An unambiguous variant identification is possible by the combination of the superposition of the experimental pole figures with the poles of the calculated variants and trace analysis of the corresponding habit planes. The predicted habit planes were determined, in particular, by a two-surface trace analysis.

The results show that the early stage of the α–γ′ transformation is comparable to the description of the α–γ′ transformation in the case of bulk-aged α-Fe. Deviations from the PTMC predictions are obtained if different γ′ variants become close to each other, if twins occur or in the case of orientation gradients. Consequently, it can be assumed that the γ′ formation in the case of bulk samples will also comply with PTMC predictions, as shown in the case of nitrided iron whiskers and bulk-aged samples, but that a more complex microstructure is formed with different self-accommodating variants.

The direct investigation of the nitrided whiskers, without changes due to sample preparation, reveals further striations on the surface of the formed γ′. These striations result from a faceting of the new surface due to a likely pronounced anisotropy of the surface energy of γ′ with low-energy {100}_γ′_ surfaces.

The great advantage of the investigation of the α–γ′ transformation under this simplified condition is that the less geometrical constraints of the iron whiskers compared with bulk samples suppress the formation of additional variants due to strain accommodation and a complex microstructure is avoided. Additionally, the starting material exhibits significantly fewer defects, no grain boundaries, and no grain growth or recrystallization in the substrate material. These benefits lead to a better understanding of the phase transformation, and the results could be used for an improved description of γ′ formation in bulk samples.

## Supplementary Material

Supporting information file. DOI: 10.1107/S1600576720005981/nb5265sup1.pdf

## Figures and Tables

**Table 1:** List of some rational b.c.c./f.c.c. orientation relationships formulated for the relation between α-Fe and γ′-Fe_4_N (see Fig. 2) as relevant for the current work

Bain†	Pitsch‡	Nishiyama–Wassermann§	Kurdjumov–Sachs¶	Mehl††
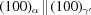	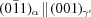	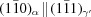	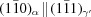	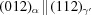
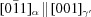	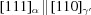	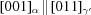	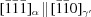	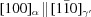
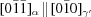	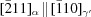	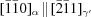	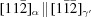	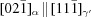

† Bain & Dunkirk (1924 ▸).

‡ Pitsch (1959*a* ▸,*b* ▸).

§ Nishiyama (1934 ▸); Wassermann (1935 ▸).

¶ Kurdjumow & Sachs (1930 ▸).

†† Mehl *et al.* (1934 ▸); Booker (1961 ▸).

**Table 2:** Transformation rules for the b.c.c. parent phase and the corresponding Bain, slip plane/direction (SP/SD) and surface group of the γ′ variants for the OR predicted by PTMC We use the numbering scheme adopted from the list of KS variants given by Morito *et al.* (2003 ▸). Each of the current variants of the PTMC OR is assigned the same variant number as the orientationally closest KS variants, which, in turn, are listed in Table S1 of the supporting information.

					Misorientation from variant 1	
Variant	Symmetry operation	Bain group	SP/SD group	Surface group	Angle (°)	Axis
1	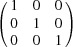	1	1	1	–	–
2	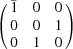	2	3	2	60.5	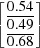
3	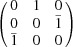	3	3	2	58.2	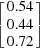
4	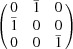	1	6	1	4.2	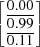
5	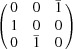	2	6	3	58.2	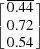
6	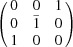	3	1	3	57.5	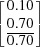
7	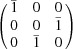	2	2	2	55.1	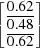
8	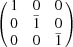	1	4	1	7.7	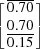
9	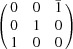	3	4	3	53.8	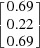
10	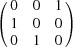	2	6	3	52.9	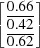
11	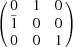	1	6	1	11.0	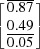
12	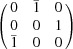	3	2	2	56.5	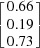
13	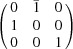	1	5	1	11.0	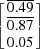
14	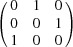	3	2	2	52.9	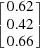
15	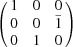	2	2	2	57.7	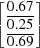
16	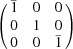	1	1	1	13.6	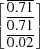
17	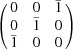	3	1	3	50.9	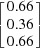
18	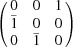	2	5	3	55.2	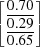
19	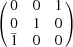	3	4	3	53.8	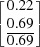
20	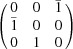	2	5	3	56.5	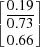
21	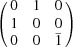	1	5	1	15.0	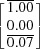
22	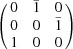	3	3	2	55.2	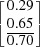
23	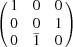	2	3	2	57.7	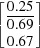
24	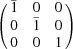	1	4	1	15.6	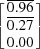
						

**Table 3:** Input parameters for the PTMC calculations and Bain distortion of variant 1

Bain correspondence	Shear plane and shear direction				Lattice parameters (Å)		Bain distortion
**K**	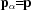	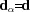	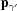	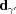	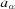	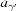	**B**
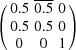	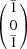	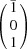	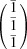	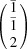	2.866†	3.795‡	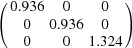
							

† Straumanis & Kim (1969 ▸).

‡ Jack (1948 ▸).

**Table 4:** Transformation matrix, habit planes and IPS for variant 1

Transformation matrix†	Habit planes		Invariant plane strain
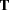	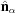 = 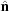	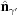	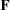
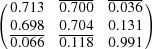	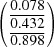	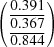	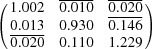
			

† In this case, the transformation matrix represents the orientation relationship between α and γ′ after shear, where each row of the matrix represents the components of a basis vector of γ′ in the coordinate system of the α-Fe matrix. The conversion of planes and vectors between the γ′ and the α lattice is then achieved according to the conversion with the correspondence matrix (Schumann, 1979 ▸).

**Table 5:** Influence of the LIS on the (001)_α_ surface normal and the corresponding γ′ planes for the variants shown in Fig. 10 ▸ (bold numbers)

Surface group	Bain group	Variant	Normal after LIS	
S			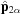	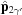
1	1	**1**, 4, **11**, 13, **16**, 21, 24	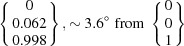	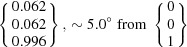
2	2/3	**2**, 3, 7, 12, 14, **15**, **22**, 23	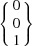	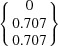
3	2/3	5, 6, 9, 10, 17, 18, **19**, 20	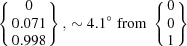	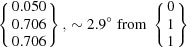
				

**Table 6:** Simplified consideration of the broken bond model for the {100}, {110} and {111} γ′-Fe_4_N planes (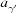: lattice parameter of γ′-Fe_4_N)

Plane	Area of unit mesh considered		No. of broken Fe—N bonds	Energy contribution Fe—N	No. of broken Fe—Fe bonds	Energy contribution Fe—Fe
{100}	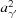		1	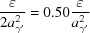	8	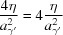
{110}	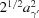		2	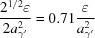	12	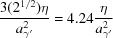
{111}	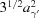		3	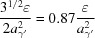	12	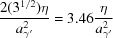

## Footnotes

1 Hence, if α′′-Fe_16_N_2_ is encountered in the diffusion zone after nitriding at typical nitriding temperatures between 773 and 853 K, this phase has definitely developed during the cooling to ambient temperature because of the decreasing solubility of nitro­gen. The same is likely for γ′-Fe_4_N precipitates in the diffusion zone.

2 Variants are different possibilities to orientate the crystal of the child phase relative to the crystal of the parent phase under consideration of the crystal symmetry of the parent phase, while maintaining the orientation relationship between parent and child phases (Nolze, 2008 ▸).

3 The most accurate method for the investigation of whiskers, and one that avoids damage of the whiskers, is a direct investigation of single whiskers on the growth substrate as described in the literature (Susan *et al.*, 2012 ▸; Stein *et al.*, 2015 ▸). However, this was not possible owing to the high density of grown whiskers on the growth substrate and the correspondingly small distances between them.

5 The new planes after shear could be calculated as  for α-Fe and  for γ′, where  is the old plane and  is the new plane with the surface modified by the LIS.
