# Supplementary material for: Crystallography of γ′-Fe4N formation in single-crystalline α-Fe whiskers
Source: J Appl Crystallogr. 2020 Jun 12;53(Pt 4):865–79. doi: 10.1107/S1600576720005981 (PMC7401782; doi:10.1107/S1600576720005981)
Supplement: Supplementary file 1 [file j-53-00865-sup1.pdf]

JOURNAL OF
APPLIED
CRYSTALLOGRAPHY

Volume 53 (2020)

Supporting information for article:

Crystallography of γ'-Fe4N formation in single-crystalline α-Fe whiskers

Helge Schumann, Gunther Richter and Andreas Leineweber

Table S1 Variants of the PTMC OR given in Appendix A together with the orientationally closest KS variants using the numbering scheme adopted from the list of KS variants given by Morito et al. (Morito et al., 2003). The close packed direction (CD) and close packed plane groups (CP) of the KS variants are formulated for the relation between α-Fe and γ'-Fe_4N.

PTMC variant	Symmetry operation	KS variant	Parallel close packed directions (CD) and close packed planes (CP)			
			CD group	$\begin{bmatrix}u\\v\\w\end{bmatrix}_\alpha \parallel \begin{bmatrix}u\\v\\w\end{bmatrix}_{\gamma'}$	CP group	$\begin{pmatrix}h\\k\\l\end{pmatrix}_\alpha \parallel \begin{pmatrix}h\\k\\l\end{pmatrix}_{\gamma'}$
1	$\begin{pmatrix}1&0&0\\0&1&0\\0&0&1\end{pmatrix}$	1	1	$\begin{bmatrix}\bar{1}\\\bar{1}\\1\end{bmatrix}_\alpha \parallel \begin{bmatrix}0\\\bar{1}\\1\end{bmatrix}_{\gamma'}$	1	$\begin{pmatrix}0\\1\\1\end{pmatrix}_\alpha \parallel \begin{pmatrix}\bar{1}\\1\\1\end{pmatrix}_{\gamma'}$
2	$\begin{pmatrix}\bar{1}&0&0\\0&0&1\\0&1&0\end{pmatrix}$	2		$\begin{bmatrix}\bar{1}\\\bar{1}\\1\end{bmatrix}_\alpha \parallel \begin{bmatrix}0\\\bar{1}\\1\end{bmatrix}_{\gamma'}$		$\begin{pmatrix}0\\1\\1\end{pmatrix}_\alpha \parallel \begin{pmatrix}1\\1\\1\end{pmatrix}_{\gamma'}$
3	$\begin{pmatrix}0&1&0\\0&0&\bar{1}\\\bar{1}&0&0\end{pmatrix}$	3		$\begin{bmatrix}\bar{1}\\\bar{1}\\1\end{bmatrix}_\alpha \parallel \begin{bmatrix}\bar{1}\\0\\1\end{bmatrix}_{\gamma'}$	2	$\begin{pmatrix}1\\0\\1\end{pmatrix}_\alpha \parallel \begin{pmatrix}1\\1\\1\end{pmatrix}_{\gamma'}$
4	$\begin{pmatrix}0&\bar{1}&0\\\bar{1}&0&0\\0&0&\bar{1}\end{pmatrix}$	4		$\begin{bmatrix}\bar{1}\\\bar{1}\\1\end{bmatrix}_\alpha \parallel \begin{bmatrix}\bar{1}\\0\\1\end{bmatrix}_{\gamma'}$		$\begin{pmatrix}1\\0\\1\end{pmatrix}_\alpha \parallel \begin{pmatrix}1\\\bar{1}\\1\end{pmatrix}_{\gamma'}$
5	$\begin{pmatrix}0&0&\bar{1}\\1&0&0\\0&\bar{1}&0\end{pmatrix}$	5		$\begin{bmatrix}\bar{1}\\\bar{1}\\1\end{bmatrix}_\alpha \parallel \begin{bmatrix}\bar{1}\\\bar{1}\\0\end{bmatrix}_{\gamma'}$	3	$\begin{pmatrix}\bar{1}\\1\\0\end{pmatrix}_\alpha \parallel \begin{pmatrix}\bar{1}\\1\\\bar{1}\end{pmatrix}_{\gamma'}$
6	$\begin{pmatrix}0&0&1\\0&\bar{1}&0\\1&0&0\end{pmatrix}$	6		$\begin{bmatrix}\bar{1}\\\bar{1}\\1\end{bmatrix}_\alpha \parallel \begin{bmatrix}\bar{1}\\\bar{1}\\0\end{bmatrix}_{\gamma'}$		$\begin{pmatrix}\bar{1}\\1\\0\end{pmatrix}_\alpha \parallel \begin{pmatrix}\bar{1}\\1\\1\end{pmatrix}_{\gamma'}$
7	$\begin{pmatrix}\bar{1}&0&0\\0&0&\bar{1}\\0&\bar{1}&0\end{pmatrix}$	7	2	$\begin{bmatrix}\bar{1}\\1\\\bar{1}\end{bmatrix}_\alpha \parallel \begin{bmatrix}0\\1\\\bar{1}\end{bmatrix}_{\gamma'}$	1	$\begin{pmatrix}0\\1\\1\end{pmatrix}_\alpha \parallel \begin{pmatrix}\bar{1}\\1\\1\end{pmatrix}_{\gamma'}$
8	$\begin{pmatrix}1&0&0\\0&\bar{1}&0\\0&0&\bar{1}\end{pmatrix}$	8		$\begin{bmatrix}\bar{1}\\1\\\bar{1}\end{bmatrix}_\alpha \parallel \begin{bmatrix}0\\1\\\bar{1}\end{bmatrix}_{\gamma'}$		$\begin{pmatrix}0\\1\\1\end{pmatrix}_\alpha \parallel \begin{pmatrix}1\\1\\1\end{pmatrix}_{\gamma'}$
9	$\begin{pmatrix}0&0&\bar{1}\\0&1&0\\1&0&0\end{pmatrix}$	9		$\begin{bmatrix}\bar{1}\\1\\\bar{1}\end{bmatrix}_\alpha \parallel \begin{bmatrix}\bar{1}\\1\\0\end{bmatrix}_{\gamma'}$	4	$\begin{pmatrix}1\\1\\0\end{pmatrix}_\alpha \parallel \begin{pmatrix}1\\1\\1\end{pmatrix}_{\gamma'}$
10	$\begin{pmatrix}0&0&1\\1&0&0\\0&1&0\end{pmatrix}$	10		$\begin{bmatrix}\bar{1}\\1\\\bar{1}\end{bmatrix}_\alpha \parallel \begin{bmatrix}\bar{1}\\1\\0\end{bmatrix}_{\gamma'}$		$\begin{pmatrix}1\\1\\0\end{pmatrix}_\alpha \parallel \begin{pmatrix}1\\1\\\bar{1}\end{pmatrix}_{\gamma'}$
11	$\begin{pmatrix}0&1&0\\\bar{1}&0&0\\0&0&1\end{pmatrix}$	11		$\begin{bmatrix}\bar{1}\\1\\\bar{1}\end{bmatrix}_\alpha \parallel \begin{bmatrix}\bar{1}\\0\\\bar{1}\end{bmatrix}_{\gamma'}$	5	$\begin{pmatrix}1\\0\\\bar{1}\end{pmatrix}_\alpha \parallel \begin{pmatrix}1\\1\\\bar{1}\end{pmatrix}_{\gamma'}$
12	$\begin{pmatrix}0&\bar{1}&0\\0&0&1\\\bar{1}&0&0\end{pmatrix}$	12		$\begin{bmatrix}\bar{1}\\1\\\bar{1}\end{bmatrix}_\alpha \parallel \begin{bmatrix}\bar{1}\\0\\\bar{1}\end{bmatrix}_{\gamma'}$		$\begin{pmatrix}1\\0\\\bar{1}\end{pmatrix}_\alpha \parallel \begin{pmatrix}1\\\bar{1}\\\bar{1}\end{pmatrix}_{\gamma'}$

13	$\begin{pmatrix}0 & \bar{1} & 0\\ 1 & 0 & 0\\ 0 & 0 & 1\end{pmatrix}$	13	3	$\begin{bmatrix}1\\ \bar{1}\\ \bar{1}\end{bmatrix}_{\alpha} \parallel \begin{bmatrix}1\\ 0\\ \bar{1}\end{bmatrix}_{\gamma'}$	2	$\begin{pmatrix}1\\ 0\\ 1\end{pmatrix}_{\alpha} \parallel \begin{pmatrix}1\\ 1\\ 1\end{pmatrix}_{\gamma'}$
14	$\begin{pmatrix}0 & 1 & 0\\ 0 & 0 & 1\\ 1 & 0 & 0\end{pmatrix}$	14		$\begin{bmatrix}1\\ \bar{1}\\ \bar{1}\end{bmatrix}_{\alpha} \parallel \begin{bmatrix}1\\ 0\\ \bar{1}\end{bmatrix}_{\gamma'}$		$\begin{pmatrix}1\\ 0\\ 1\end{pmatrix}_{\alpha} \parallel \begin{pmatrix}1\\ \bar{1}\\ 1\end{pmatrix}_{\gamma'}$
15	$\begin{pmatrix}1 & 0 & 0\\ 0 & 0 & \bar{1}\\ 0 & 1 & 0\end{pmatrix}$	15		$\begin{bmatrix}1\\ \bar{1}\\ \bar{1}\end{bmatrix}_{\alpha} \parallel \begin{bmatrix}0\\ \bar{1}\\ \bar{1}\end{bmatrix}_{\gamma'}$	6	$\begin{pmatrix}0\\ \bar{1}\\ 1\end{pmatrix}_{\alpha} \parallel \begin{pmatrix}1\\ \bar{1}\\ 1\end{pmatrix}_{\gamma'}$
16	$\begin{pmatrix}\bar{1} & 0 & 0\\ 0 & 1 & 0\\ 0 & 0 & \bar{1}\end{pmatrix}$	16		$\begin{bmatrix}1\\ \bar{1}\\ \bar{1}\end{bmatrix}_{\alpha} \parallel \begin{bmatrix}0\\ \bar{1}\\ \bar{1}\end{bmatrix}_{\gamma'}$		$\begin{pmatrix}0\\ \bar{1}\\ 1\end{pmatrix}_{\alpha} \parallel \begin{pmatrix}\bar{1}\\ \bar{1}\\ 1\end{pmatrix}_{\gamma'}$
17	$\begin{pmatrix}0 & 0 & \bar{1}\\ 0 & \bar{1} & 0\\ \bar{1} & 0 & 0\end{pmatrix}$	17		$\begin{bmatrix}1\\ \bar{1}\\ \bar{1}\end{bmatrix}_{\alpha} \parallel \begin{bmatrix}1\\ \bar{1}\\ 0\end{bmatrix}_{\gamma'}$	4	$\begin{pmatrix}1\\ 1\\ 0\end{pmatrix}_{\alpha} \parallel \begin{pmatrix}1\\ 1\\ \bar{1}\end{pmatrix}_{\gamma'}$
18	$\begin{pmatrix}0 & 0 & 1\\ \bar{1} & 0 & 0\\ 0 & \bar{1} & 0\end{pmatrix}$	18		$\begin{bmatrix}1\\ \bar{1}\\ \bar{1}\end{bmatrix}_{\alpha} \parallel \begin{bmatrix}1\\ \bar{1}\\ 0\end{bmatrix}_{\gamma'}$		$\begin{pmatrix}1\\ 1\\ 0\end{pmatrix}_{\alpha} \parallel \begin{pmatrix}1\\ 1\\ 1\end{pmatrix}_{\gamma'}$
19	$\begin{pmatrix}0 & 0 & 1\\ 0 & 1 & 0\\ \bar{1} & 0 & 0\end{pmatrix}$	19	4	$\begin{bmatrix}1\\ 1\\ 1\end{bmatrix}_{\alpha} \parallel \begin{bmatrix}1\\ 1\\ 0\end{bmatrix}_{\gamma'}$	3	$\begin{pmatrix}\bar{1}\\ 1\\ 0\end{pmatrix}_{\alpha} \parallel \begin{pmatrix}\bar{1}\\ 1\\ \bar{1}\end{pmatrix}_{\gamma'}$
20	$\begin{pmatrix}0 & 0 & \bar{1}\\ \bar{1} & 0 & 0\\ 0 & 1 & 0\end{pmatrix}$	20		$\begin{bmatrix}1\\ 1\\ 1\end{bmatrix}_{\alpha} \parallel \begin{bmatrix}1\\ 1\\ 0\end{bmatrix}_{\gamma'}$		$\begin{pmatrix}\bar{1}\\ 1\\ 0\end{pmatrix}_{\alpha} \parallel \begin{pmatrix}\bar{1}\\ 1\\ 1\end{pmatrix}_{\gamma'}$
21	$\begin{pmatrix}0 & 1 & 0\\ 1 & 0 & 0\\ 0 & 0 & \bar{1}\end{pmatrix}$	21		$\begin{bmatrix}1\\ 1\\ 1\end{bmatrix}_{\alpha} \parallel \begin{bmatrix}1\\ 0\\ 1\end{bmatrix}_{\gamma'}$	5	$\begin{pmatrix}1\\ 0\\ \bar{1}\end{pmatrix}_{\alpha} \parallel \begin{pmatrix}1\\ \bar{1}\\ \bar{1}\end{pmatrix}_{\gamma'}$
22	$\begin{pmatrix}0 & \bar{1} & 0\\ 0 & 0 & \bar{1}\\ 1 & 0 & 0\end{pmatrix}$	22		$\begin{bmatrix}1\\ 1\\ 1\end{bmatrix}_{\alpha} \parallel \begin{bmatrix}1\\ 0\\ 1\end{bmatrix}_{\gamma'}$		$\begin{pmatrix}1\\ 0\\ \bar{1}\end{pmatrix}_{\alpha} \parallel \begin{pmatrix}1\\ 1\\ \bar{1}\end{pmatrix}_{\gamma'}$
23	$\begin{pmatrix}1 & 0 & 0\\ 0 & 0 & 1\\ 0 & \bar{1} & 0\end{pmatrix}$	23		$\begin{bmatrix}1\\ 1\\ 1\end{bmatrix}_{\alpha} \parallel \begin{bmatrix}0\\ 1\\ 1\end{bmatrix}_{\gamma'}$	6	$\begin{pmatrix}0\\ \bar{1}\\ 1\end{pmatrix}_{\alpha} \parallel \begin{pmatrix}\bar{1}\\ \bar{1}\\ 1\end{pmatrix}_{\gamma'}$
24	$\begin{pmatrix}\bar{1} & 0 & 0\\ 0 & \bar{1} & 0\\ 0 & 0 & 1\end{pmatrix}$	24		$\begin{bmatrix}1\\ 1\\ 1\end{bmatrix}_{\alpha} \parallel \begin{bmatrix}0\\ 1\\ 1\end{bmatrix}_{\gamma'}$		$\begin{pmatrix}0\\ \bar{1}\\ 1\end{pmatrix}_{\alpha} \parallel \begin{pmatrix}1\\ \bar{1}\\ 1\end{pmatrix}_{\gamma'}$
